# Media portrayals of assisted suicide before, during, and after legalization changes: content analysis of the reporting in Austrian newspapers

**DOI:** 10.3389/fpsyt.2025.1617602

**Published:** 2025-09-18

**Authors:** Paul Pürcher, Benedikt Till, Thomas Niederkrotenthaler

**Affiliations:** ^1^ Department of Medical Psychology, Center for Public Health, Medical University of Vienna, Vienna, Austria; ^2^ Unit Public Mental Health Research, Department of Social and Preventive Medicine, Center for Public Health, Medical University of Vienna, Vienna, Austria; ^3^ Wiener Werkstaette for Suicide Research, Vienna, Austria

**Keywords:** assisted suicide, print media, media guidelines on suicide reporting, media portrayal of suicide, content analysis

## Abstract

**Introduction:**

Assisted suicide (AS) was legalized in Austria in 2022 for adults in specific circumstances, adding Austria to a list of several countries where AS has recently been legalized. The topic has been discussed controversially in the Austrian public, which has been reflected in the media reporting. Information about the content of media reports on AS is currently lacking although it is important from a suicide prevention perspective. The aim of this study was to analyze newspaper media items on AS from Austrian daily newspapers based on media guidelines for the reporting on suicide and to adapt them in the process to the specific topic of AS.

**Methods:**

Media items from 11 Austrian daily newspapers from the time period 2017 to 2022 were retrieved based on 12 predefined keywords. A total of *n* = 906 articles were analyzed with regard to 12 characteristics advised against in media guidelines and 7 recommended characteristics. We compared the quality of media items between three time periods: period 1 (before the initiation of legislation change regarding AS in Austria, 01/2017–12/2019), period 2 (around the decision of legalization of AS, 01/2020–12/2021), and period 3 (after the implementation of AS in Austria, 01/2022–12/2022).

**Results:**

Several characteristics advised against in suicide reporting guidelines (e.g., romanticization/glorification of AS) were relatively frequent across all three time periods, while recommended characteristics (e.g., references to mental health services) were less common. Comparison across time showed that stigmatization and romanticization/glorification, though prevalent, declined after AS was implemented, whereas reporting on epidemics and waves of AS peaked immediately before its implementation.

**Discussion:**

This content analysis revealed distinct patterns in media reporting on AS and showed that reports were less aligned with media guidelines than previous analyses on non-assisted suicide. Some patterns identified across all time periods, particularly tendencies to stigmatize or romanticize AS before the legislation, likely reflect polarized public attitudes, which warrant attention in education efforts. This research highlights the importance of specific aspects of media guidelines during public debate on AS and the need to tailor them to this topic.

## Introduction

Assisted suicide (AS) is defined as “the practice of providing a competent patient with a prescription for medication for the patient to use with the primary intention of ending his or her own life” (1). Sometimes, other terms or labels are used in public discourse, such as assisted death, killing on request, voluntary dying, or euthanasia (2–4), although not all of these terms carry the same meaning. Particularly, killing on request has a conceptually different meaning from AS, because it involves the active ending of the life of an individual. Voluntary death does not include the assisting component of AS, and is therefore misleading.

AS is currently legal in several countries, including the Netherlands, Belgium, Luxembourg, Spain, Germany, Portugal, Switzerland, Colombia, Canada, and New Zealand, as well as some states of the USA and Australia (5). Some of these countries and regions have changed their legislation on AS quite recently, including Spain in 2021, Germany in 2020, New Zealand in 2020, Portugal in 2023, and some Australian states in 2024. Several countries currently consider similar legislation changes or are in the process of regulating AS (e.g., Italy; for a current overview on the legal status of AS, see https://en.wikipedia.org/wiki/Assisted_suicide#Legality_by_country_and_region) (5). In Austria, the Constitutional Court of Justice legalized AS in December 2020, leaving a time frame until 31 December 2021 for the government to define and implement process-related rules and regulations for AS. As a result, AS became available to adults in Austria meeting the following criteria: suffering from an incurable, terminal illness or from a severe, chronic illness with persistent symptoms, the consequences of which permanently impair the person’s entire way of life (§6 StVfG: Death provision) (6). The process requires the setup of a death provision [*Sterbeverfügung*], which involves consultations of two physicians (with one of them being specialized in palliative care). A general 12-week waiting period can be shortened to 2 weeks in case of terminal illness. After that period, a lethal medication can be obtained from a pharmacy (7, 8).

AS has been a highly controversial topic in Austria and in many other countries considering similar legislation changes (9, 10). Debates typically focus on views about the moral implications and potential effects of AS (e.g., possible pressure on the elderly or disabled family members to die from AS), but also on specific requirements for conducting AS, the roles of suicide prevention and palliative care, and the protection of third parties (7, 11). These controversies are reflected in the public discourse and are perpetuated by the media, which both shape and reflect views and opinions about issues of societal interest (12).

In the context of media reporting on AS, it has been noted in Austria and Germany that AS was often discussed with a tendency to romanticize AS or portray it as the only option to individuals experiencing existential suffering in the context of illness (11). In contrast, other media items might stigmatize individuals considering or opting for AS or providing support in AS. Such aspects can be problematic in terms of public mental health efforts to educate the public including individuals who might consider AS. Tendencies to glorify or romanticize suicide or AS are a matter of concern, because they might provide an inaccurate and incomplete picture of the process, particularly if AS is highlighted as the only option available for individuals facing serious chronic illness. Stigmatization is problematic because of the known harms of stigma on individuals to speak about their problems and their death wishes, with negative impacts on making informed and independent decisions.

The reporting on assisted suicide is very important when it comes to both shaping and reflecting public opinions on this topic of high societal interest, but a structured content analytic work on the reporting of AS from a suicide prevention perspective is currently entirely missing. Such information is needed to inform public debates with tailored information. For the reporting on suicide in general, national public health agencies and the World Health Organization (WHO) have developed media guidelines for the reporting in order to educate media professionals about the complexities of suicide and to improve the quality of the reporting (8, 13). These guidelines specifically emphasize that romanticization, gross simplifications in the portrayal of motivations, and reasons for suicide, as well as a lack of information on helplines and organizations providing mental health support, can leave the public misinformed or unaware of the full variety of options, including the potential alternatives to suicide. The primary aim of these guidelines is to reduce the risk of additional suicides, the so-called Werther effect. Further goals are to provide more accurate information on the topic of suicide and its prevention, for example, by highlighting the complexity of any suicidal act and the reduction of stigmatization of individuals who are suicidal, or died of suicide, as well as their families and friends. When it comes to the topic of AS, the media guidelines for reporting on suicide need to be somewhat reframed and tailored to better address the topic of AS. There are several important differences between non-assisted suicide and AS that are of particular relevance when it comes to the topic of media reporting of AS and its effects. Specifically, increases in suicides after media reports on non-assisted suicide are generally a negative health outcome, and the guidelines aim to mitigate this risk. This does not *per se* apply to the topic of AS, which is now a new option to individuals under specific circumstances in some countries (including Austria). An increase in AS might reflect that some individuals made an informed decision rather than suggesting a negative health outcome. There is consensus in suicide prevention that AS is sometimes a valid option, but AS should not, however, constitute the first, but an additional last option to individuals experiencing existential suffering. Secondary goals of the media guidelines, i.e., to help ensure that the public is well-informed about AS (including any alternatives to AS), to avoid any gross simplifications, and to prevent stigmatization of individuals seeking AS, their families, and of individuals providing assistance in this process or trying to provide alternative options, apply to AS in a similar way as to (non-assisted) suicide.

With regard to non-assisted suicide, several studies have analyzed news media contents in order to investigate the degree of consistency of media reports with media guidelines (14–23). For AS, however, such analyses are entirely lacking. This is in spite of the fact that some recent versions of the guidelines highlight aspects that are potentially relevant also to AS (8). In order to provide tailored information addressing any blind spots and misconceptions in current portrayals to the public and to the media, systematic analyses of the reporting on AS are needed, particularly during times of strong public attention to the topic. Importantly, reporting characteristics likely vary depending on the legal status of AS in a given country.

The aim of this study was therefore to analyze content characteristics of newspaper media items about AS with a content analysis that was based on media guidelines for the reporting on suicide (12). In order to tailor media guidelines better to this purpose, we made adaptations to the framing of the specific recommendations, and it was necessary to adapt some coding definitions and add some new codes and coding categories of potential interest to the coding scheme. Once this process was finalized, we assessed reporting contents during the time period before legislation change, during legislation change (i.e., in the time period between the announcement of the Constitutional Court of Justice and the implementation), and the period immediately after the implementation of the legislation.

With many countries having quite recently implemented legislation changes and others considering similar changes or how to implement them, systematic analyses about reporting of AS during these phases might reveal important insight into aspects that require consideration when informing the public about AS in these different phases of the debate and legislation changes.

We assumed that there would be differences in media coverage of AS depending on the specific phase in which the media items were published. In particular, for phase 1 (before the decision of the Constitutional Court of Justice), we expected to see mainly reports about AS in other countries, most importantly Switzerland and the Netherlands, as both of these countries had a long history of legal options for AS (5). Furthermore, some media reports focused on Austrians travelling to these countries to carry out AS. We expected the bulk of heated discussions about the topic in phase 2, i.e., the period after the decision of the court and before the implementation of the legislation. At that stage, all of the details related to the legal process were negotiated, and there was a strong public interest in the topic. For phase 3, after the implementation, we expected to see more individual reports about individuals who died by AS, including some individuals who had fought for AS for themselves. As this is the first analysis that assesses media reporting on AS in a structured way, we were not able to provide specific hypotheses about how these changes over time might be reflected in the specific coding categories, but we aimed to assess this as an exploratory research question to inform hypothesis-building and replications in future analyses in other countries and media settings.

### Positionality of this research in the context of AS

This analysis is not intended to pursue a pro-AS or against-AS standpoint, but aims to shed light on some important aspects of the current reporting practices of media reporting related to this topic. Balanced reporting on AS and related aspects is important to help ensure informed decision-making where AS is one but not the only option for individuals meeting specific conditions. Stigmatization of individuals seeking AS or supporting them needs to be prevented due to the known negative effects of stigma on the disclosure of suicidal wishes and harm to those bereaved by the death or involved in the preparations for AS. Romanticization needs to be addressed as well as this might result in misinformation. Coding categories were carefully adapted to be better tailored to the topic of AS in order to prevent any overgeneralization of suicide reporting guidelines to the topic of AS.

## Materials and methods

We conducted a content analysis of *n* = 906 newspaper media items on AS published in 11 national daily newspapers in Austria between 01/2017 and 12/2022. We selected this time period based on a change of legislation concerning AS in Austria in 2022. The 6 years of interest were divided into three periods: a time period well before the legalization change and the bulk of related public discussions (period 1, 01/2017–12/2019), a time period shortly before and after the verdict of the Constitutional Court of Justice (period 2, 01/2020–12/2021), and the first year of implementation of AS (period 3, 01/2022–12/2022). Regarding our decision to focus on print media items, it is important to note that newspaper reports are still a major source of information in the Austrian population, and 51.2% of Austrians regularly read newspapers (24). We selected newspapers with national coverage based on sales figures and overall included 11 print media with national reach and the highest sales numbers (24).

### Sample and search terms

All *n* = 906 print media items on AS published in the 11 Austrian daily newspapers (see the coding scheme in **Supplementary Table S1** for a list of all included newspapers) were downloaded from the Austrian Press Agency (APA) news database. We searched the database with a set of 12 predefined keywords that reflect the most common words and terms used in reference to AS: *Sterbehilfe* [“help to die,” a term for AS], *Sterbeverfügung* [“death provision”], Selbsttötung* [“self-killing”], “*Tötung auf Verlangen*” [“killing on request,” used for “active euthanasia”], “*Beihilfe zum Suizid*” [“assistance to suicide”], “*Beihilfe zum Selbstmord*” [“assistance to self-murder”], “*Beihilfe zum Freitod*” [“assistance to free/voluntary death”], “*Beihilfe zur Selbsttötung*” [“assistance to self-killing”], “*Assistierter Suizid*” [“assisted suicide”], “*Assistierter Selbstmord*” [“assisted self-murder”], “*Assistierter Freitod*” [“assisted free/voluntary death”], and “*Assistierte Selbsttötung*” [“assisted self-killing”]. Note that in German-speaking countries, the word “suicide” is referred to with a variety of different words, including “Suizid” [“suicide”], “Freitod” [“free/voluntary death”], “Selbstmord” [“self-murder”], and “Selbsttötung” [“self-killing”]. Some of the translations do not reflect accurate English, but we highlight them here as they provide the connotation related to each of the terms. All of these keywords were used for media item searches in order not to miss any relevant media items.

**Figure 1:**
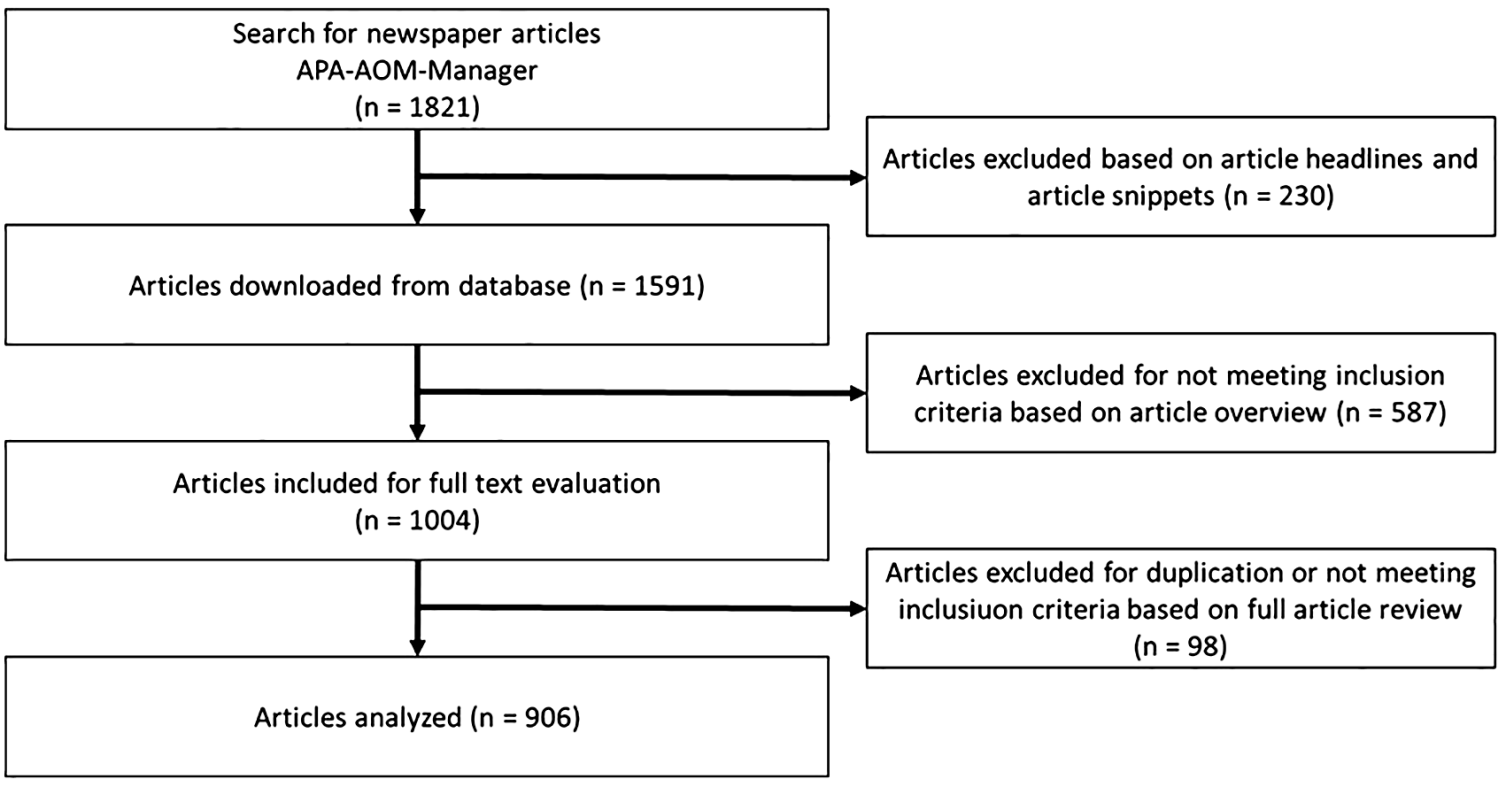
Flowchart of the media item selection process.

The study flowchart is shown in **Figure 1**. In the first step, *n* = 1,821 media items were retrieved. We excluded *n* = 230 media items that were not relevant based on screening headings and snippets. In the next step, we screened full texts to exclude items that did not have a major focus on AS-related content. In order to have a major focus on AS, at least one of the three following requirements needed to be applied: 1) At least 50% of the text were focused on the topic of AS; 2) at least three different keywords from the keyword list were used in the main text; or 3) at least one of these keywords was used in the headline. In addition, if criterion 3 applied, the media items also had to include at least one full paragraph on AS.

We came up with this definition based on careful screening of media items in order not to miss any media item that might have a focus on AS. Of note, if we had only used the first criterion, we would have missed some items that focused on AS in a smaller proportion of the text, but raised the topic repeatedly (criterion 2) or highlighted its relevance in the headline, which is also relevant to defining the focus of an article, even if the main text dedicates less than 50% to the topic area (criterion 3).

Event announcements and fictional media items were excluded. This process resulted in *n* = 1,004 media items that were included in the full-text evaluation. After removing duplicates and further media items that did not fulfil the inclusion criteria, *n* = 906 media items were retained and included in the content analysis.

### Coding categories and codes

The coding categories used for this content analysis were based on media guidelines for suicide reporting and prior content analyses for suicide-related media contents (8, 13, 18). In total, 40 coding categories, including 21 for general characteristics (e.g., publication date, content focus), 12 for characteristics that are advised against in media guidelines (e.g., presence of romanticizing content), and 7 for recommended characteristics (e.g., reference to crisis counseling), were used for the analysis.

Most, but not all, of these coding categories were consistent with media guidelines for suicide reporting as well as with previous content analytic work that was based on these guidelines (18). Some changes and deviations from the original codings for (non-assisted) suicide that were deemed necessary are described below.

Media guidelines generally group code categories into characteristics that are recommended and advised against for the media reporting of suicide (8, 13). The different code categories were accordingly grouped into “recommended in media guidelines” as well as “advised against in media guidelines.” For the present analysis, we added a few more code categories, such as “general characteristics.” These characteristics were deemed helpful to describe the reporting but would not immediately fall into a “recommended” or “advised against” group.

### Coding procedure and adaptations of the codebook

The coding process is visualized in **Supplementary Figure S1** in the **Supplementary Material** (overview of the coding process). The construction of an appropriate codebook for this analysis involved several steps: We used the codebook from a previous content analysis on (non-assisted) suicide reporting as a basis for the development of the coding scheme (18). In accordance with (25), this was the deductive aspect of the analysis. Media items were screened by the first and last authors for any adaptations and additions that were deemed necessary to capture specific aspects of the primary material about AS. Several potential needs for adaptations to the codebook were identified and discussed, and if there was consensus, they were implemented. Changes mainly reflected minor adaptations of existing coding (18). We explain some important basic aspects of selected code definitions in the following paragraphs. **Supplementary Table S1** in the **Supplementary Materials** provides further elaborations on all codes and coding categories as well as their definitions that we have used in this analysis.

In terms of *type of media item*, we coded each item as either news (i.e., items disclosing new timely developments that were just revealed), background items (i.e., items that relate to recent news, normally aiming to explore the possible impact or meaning beyond the news reporting), comments (opinions of readers or the editor), interviews, or other text types.

Because we found that the *focus areas* of items were different from items on non-assisted suicide, we developed new codes to capture the focus areas of media items. Specifically, we assessed if the item was focusing on the preparations for AS (i.e., any aspects of the process needed before the actual AS can be carried out including the setting up of a death provision), the procedure of actually carrying out the AS after these preparations, postvention in the context of AS, palliative care in relation to AS, suicide prevention in the context of AS, and/or if a focus was on general reflections about AS.

Some specific adaptations were made to the coding category *stigmatization* of AS in comparison to the previous code used for non-assisted suicide (18). Stigmatization is part of the group of characteristics advised against in media recommendations. In order to reflect the media items on AS, we added that any direct comparisons of discussions about AS with the crime of euthanasia during WW2 in Austria that would make it appear as if motivations were similar would qualify as stigmatizing. Furthermore, it was deemed necessary to include a statement that objections to AS or the process implemented to carry it out without any stigmatizing wording would not qualify as stigmatization. A list of words that were often used to stigmatize individuals was collected from media guidelines on the reduction of stigma in media reporting on mental health, which some of the authors have co-authored (26).

We also needed to make some specifications regarding the code category *romanticization/glorification* used for (non-assisted) suicide. This coding category was coded positive if there was any aspect in the wording that might glorify or romanticize the phenomenon of AS, individuals considering AS or dying by AS, or portray them as a hero, even if this was implicit or subtle. Among others, terminology such as “dying with dignity” qualified as romanticization if it was used as a generic term for AS.

At the in-depth screening stage, we also added some codes and coding categories because of content that was not represented in items about non-assisted suicide but appeared to play a major role in the reporting of AS. This was the inductive part of the content analysis (25). For example, considerations related to the portrayed perceived appropriateness of the law and related processes (coding category “Difficulty level to get AS approved or to carry out AS”; AS portrayed as “too easy to” or “too difficult to carry out” versus “no clear valuation”) and the provision of contacts that can assist in the planning or conduction of AS were added to the coding categories. Furthermore, a code reflecting motivations for considering AS as well as focus areas of media items related to AS, specifically a focus on preparations for AS (e.g., to set up a death provision and get it approved), the procedure of carrying out the AS once preparations were finalized, a focus on palliative care or suicide prevention in the context of AS, and a focus on general reflections about AS were added in that process. Furthermore, we added coding categories to capture if the specific topic of “killing on request”—which is distinct from AS in the sense used here—was discussed and the names of the countries that were brought up in the media item (i.e., specific countries that were discussed with regard to their legislation on AS). Finally, we added a coding category to assess if the topic of social pressure on vulnerable groups to consider or opt for AS in the face of suffering was mentioned in the media item.

After the initial phase of screening and adaptation of the codebook, the main coder (the first author) coded a selected sample of media items for training from across the entire period. Uncertain cases were discussed with the senior author and resolved. Only minor adaptations of the codebook were made at that point. Subsequently, a random sample of *n* = 43 media items not included in the training phase (5% of included media items) was selected for intercoder reliability testing. This approach was consistent with previous content analyses of suicide-related media items (e.g., 21, 22). Author 1 and author 3 coded the sample of items independently from each other, and the percentage agreement and Krippendorff’s alpha were calculated with the online tool ReCal2 reliability for each coding category (27). Percentage agreement of coding categories ranged from 95% to 100%, and Krippendorff’s alpha was >0.80 for all coding categories, which indicates very good reliability (28, 29). An overview of the coding procedure is provided in the **Supplementary Material** (see **Supplementary Figure S1**, **Supplementary Materials**, page 17).

### Data analysis

We grouped the retrieved *n* = 906 media items (2017: *n* = 21 media items, 2018: *n* = 40 media items, 2019: *n* = 42 media items, 2020: *n* = 373 media items, 2021: *n* = 346 media items, 2022: *n* = 84 media items) into three time periods: period 1 (*n* = 103 media items, 2017–2019), representing the time period prior to the legislation change; period 2 (*n* = 719, 2020–2021), representing the time period around the decision of the Austrian Constitutional Court of Justice to legalize AS in December 2020 and before its actual legalization; and period 3 (*n* = 84, 2022), representing the time period immediately after the implementation and thus practical availability of AS on 1 January 2022.

In order to compare frequencies of media item characteristics between the three time periods, chi-squared tests were calculated. For small cell counts (<5), Fisher’s exact tests were used (30).

## Results

Among the *n* = 906 media items, *n* = 234 (25.8%) were news items, *n* = 267 (29.5%) were background information items, *n* = 320 (35.3%) were reader and editorial comments, *n* = 56 (6.2%) were interviews, and *n* = 29 (3.2%) were other types of items. *N* = 36 media items (4.0%) focused on one or more specific individual person(s) without any general aspects about AS, *n* = 727 media items (80.2%) had a general (non-personal) focus, and *n* = 143 media items (16.8%) were mixed in terms of personal and general focus.

Over all three periods, any illness (unspecified) was the most prevalent motivation for AS (*n* = 364, 40.2%), followed by human/personal rights (*n* = 196, 21.6%), somatic diseases (*n* = 172, 19.0%), and pain and suffering (*n* = 140, 15.5%). *N* = 262 media items (28.9%) reported no specific motivation for AS. Most reports focused on the situation or cases in Austria (*n* = 802, 88.5%), followed by Switzerland (*n* = 193, 21.3%) and Germany (*n* = 169, 18.7%), with foreign country situations being most frequently reported in phase 1, before the legislation change.

A majority of media items included a citation of experts (*n* = 550, 60.7%), mainly of politicians (*n* = 143, 26.0%), legal experts (*n* = 89, 16.2%), medical doctors other than a psychiatrist, palliative care physician, or unspecified MDs (*n* = 52, 9,5%), and palliative care physicians (*n* = 52, 9.5%). Experts often had neutral or mixed views toward AS (*n* = 205, 37.3%) or were against AS (*n* = 158, 28.7%), but considerably less frequently voiced sole support for AS (*n* = 87, 15.8%). Citations from family and friends were rare (*n* = 46, 5.1%), as well as any reported effects of AS on family and friends (*n* = 127, 14.0%). If citations from them were included, these often were in favor of AS (*n* = 28, 60.9%). Statistical data were provided in 23.6% of the media items (*n* = 214).

Across the entire observation period (2017–2022), several coding categories advised against in media guidelines were relatively common. These included tendencies of romanticization or glorification of AS (*n* = 464, 51.2%), the inclusion of false myths about suicide (*n* = 325, 35.9%), and stigmatizing language (*n* = 304, 33.6%). Furthermore, *n* = 188 media items (20.8%) portrayed AS as inevitable and the only option to deal with existential suffering. The latter finding on inevitability is particularly concerning, because as per our definition of inevitability, there was no single indication in these media items about any doubt or consideration of alternative options at any time of the process leading up to AS. Correct and balanced information, however, is key to fully informed decision-making.

Among the coding categories that were recommended in media guidelines, many coding categories had a low prevalence across the entire observation. For example, a reference to crisis intervention services, counseling, or mental health treatment was included in *n* = 34 items (3.7%). Only very few items linked AS to crisis situations or mental health problems (*n* = 91, 10.0%), and only a small proportion reported about individuals or situations where consideration of AS resulted in a different outcome than AS (*n* = 13, 1.4%). Overall, *n* = 392 items (43.3%) included at least one alternative to AS, and there were no media items that focused on the recovery and healing of individuals bereaved by AS. See **Table 1** for an overview of the frequencies of coding categories of general characteristics in the study periods.

**Table 1:** Reporting of general characteristics before, during, and after legislation changes legalizing AS.

Coding categories		Total *n* (all 3 periods) (*n* = 906)	Period 1 (2017–2019) (*n* = 103)	Period 2 (2020–2021) (*n* = 719)	Period 3 (2022) (*n* = 84)	Comparison of period 1 vs. 2^1^	Comparison of period 2 vs. 3^1^
General Characteristics							
Type of content	*News*	234 (25.8)	44 (42.7)	167 (23.2)	23 (27.4)	17.94^***^	0.72
	*Background*	267 (29.5)	28 (27.2)	204 (28.4)	35 (41.7)	0.06	6.36^*^
	*Comment (reader’s view/editor’s view)*	320 (35.3)	20 (19.4)	282 (39.2)	18 (21.4)	15.20^***^	10.17^**^
	*Interview*	56 (6.2)	6 (5.8)	44 (6.1)	6 (7.1)	0.01	0.14
	*Other*	29 (3.2)	5 (4.9)	22 (3.1)	2 (2.4)	n/a	n/a
Killing on request		399 (44.0)	50 (48.5)	322 (44.8)	27 (32.1)	0.51	4.89*
Article focus (individual vs. general)	*Individual focus*	36 (4.0)	11 (10.7)	11 (1.5)	14 (16.7)	n/a^***^	n/a^***^
	*General focus*	727 (80.2)	48 (46.6)	611 (85.0)	68 (81.0)	83.47^***^	0.93
	*Mixed focus*	143 (15.8)	44 (42.7)	97 (13.5)	2 (2.4)	54.16^***^	8.59^**^
Description of the character of a person considering AS or dying by AS (*n* = 179)	*Good*	77 (43.0)	26 (47.3)	38 (35.2)	13 (81.3)	2.23	12.21^***^
	*Bad*	0 (0.0)	0 (0.0)	0 (0.0)	0 (0.0)	–	–
	*Mixed*	102 (57.0)	29 (52.7)	70 (64.8)	3 (18.8)	2.23	12.21^***^
Content focus	*Preparation of AS*	215 (23.7)	33 (32.0)	149 (20.7)	33 (39.3)	6.62^*^	14.79^***^
	*Procedure of carrying out AS*	158 (17.4)	31 (30.1)	106 (14.7)	21 (25.0)	15.29^***^	5.94^*^
	*Postvention of AS*	49 (5.4)	24 (23.3)	25 (3.5)	0 (0.0)	36.16^***^	n/a
	*Palliative care*	301 (33.2)	20 (19.4)	249 (34.6)	32 (38.1)	9.47^**^	0.40
	*Suicide prevention*	46 (5.1)	2 (1.9)	38 (5.3)	6 (7.1)	2.18	n/a
	*Reflection (pro/con AS)*	868 (95.8)	89 (86.4)	708 (98.5)	71 (84.5)	n/a^***^	n/a^***^
	*Other*	227 (25.1)	28 (27.2)	186 (25.9)	13 (15.5)	0.08	4.36^*^
Motivations	*Any illness, unspecified*	364 (40.2)	34 (33.0)	301 (41.9)	29 (34.5)	2.93	1.67
	*Mental illness*	65 (7.2)	15 (14.6)	49 (6.8)	1 (1.2)	7.53^*^	4.08^*^
	*Somatic disease*	172 (19.0)	32 (31.1)	113 (15.7)	27 (32.1)	14.62^***^	14.10^***^
	*Dementia*	39 (4.3)	15 (14.6)	23 (3.2)	1 (1.2)	n/a^***^	n/a
	*Age/life weariness*	87 (9.6)	15 (14.6)	69 (9.6)	3 (3.6)	2.42	3.35
	*Human rights/personal rights*	196 (21.6)	24 (23.3)	167 (23.2)	5 (6.0)	0.00	13.33^***^
	*Isolation/loneliness*	30 (3.3)	2 (1.9)	28 (3.9)	0 (0.0)	n/a	n/a
	*Burden to others*	54 (6.0)	2 (1.9)	50 (7.0)	2 (2.4)	3.82	2.60
	*Alternative to non-assisted suicide*	12 (1.3)	6 (5.8)	6 (0.8)	0 (0.0)	n/a^**^	n/a
	*Pain & suffering*	140 (15.5)	27 (26.2)	109 (15.2)	4 (4.8)	7.97**	6.73*
	*Other*	129 (14.2)	21 (20.4)	106 (14.7)	2(2.4)	2.20	9.87^**^
	*No motivation*	262 (28.9)	12 (11.7)	221 (30.7)	29 (34.5)	16.16^***^	0.50
Country situations	*Austria*	802 (88.5)	64 (62.1)	667 (92.8)	71 (84.5)	85.87^***^	6.87^**^
	*Germany*	169 (18.7)	13 (12.6)	150 (20.9)	6 (7.1)	3.85	9.04^**^
	*Switzerland*	193 (21.3)	35 (34.0)	142 (19.7)	16 (19.0)	10.80^**^	0.02
	*Belgium*	109 (12.0)	21 (20.4)	81 (11.3)	7 (8.3)	6.90^**^	0.66
	*Netherlands*	155 (17.1)	36 (35.0)	109 (15.2)	10 (11.9)	24.29^***^	0.63
	*Spain*	34 (3.8)	1 (1.0)	30 (4.2)	3 (3.6)	n/a	n/a
	*Portugal*	18 (2.0)	0 (0.0)	17 (2.4)	1 (1.2)	n/a	n/a
	*Italy*	12 (1.3)	1 (1.0)	7 (1.0)	4 (4.8)	n/a	7.99^**^
	*Other country*	164 (18.1)	33 (32.0)	116 (16.1)	15 (17.9)	15.36^***^	0.16
	*No* sp*ecific country*	5 (0.6)	3 (2.9)	1 (0.1)	1 (1.2)	n/a^**^	n/a
Interview/citation of expert		550 (60.7)	54 (52.4)	441 (61.3)	55 (65.5)	2.98	0.55
Type of expert (*n* = 550)	*Representative of an ethical body*	14 (2.5)	0 (0.0)	13 (2.9)	1 (1.8)	n/a	n/a
	*Legal expert*	89 (16.2)	9 (16.7)	67 (15.2)	13 (23.6)	0.08	2.58
	*Medical doctor other than psychiatrist/palliative care physician or unspecified*	52 (9.5)	10 (18.5)	34 (7.7)	8 (14.5)	n/a^*^	n/a
	*Psychiatrist*	12 (2.2)	1 (1.9)	9 (2.0)	2 (3.6)	n/a	n/a
	*Palliative care physician*	52 (9.5)	5 (9.3)	35 (7.9)	12 (21.8)	n/a	10.99^***^
	*Suicide assistant not from an organization*	3 (0.5)	1 (1.9)	2 (0.5)	0 (0.0)	n/a	n/a
	*Psychologist/psychotherapist*	9 (1.6)	2 (3.7)	7 (1.6)	0 (0.0)	n/a	n/a
	*Suicide organization*	42 (7.6)	0 (0.0)	32 (7.3)	10 (18.2)	n/a^*^	n/a^*^
	*Funeral director*	1 (0.2)	0 (0.0)	0 (0.0)	1 (1.8)	–	n/a
	*Author/editor/media professional*	17 (3.1)	1 (1.9)	15 (3.4)	1 (1.8)	n/a	n/a
	*Politician*	143 (26.0)	12 (22.2)	125 (28.3)	6 (10.9)	0.90	7.65^**^
	*Others or unspecified*	357 (64.9)	33 (61.1)	287 (65.1)	37 (67.3)	0.33	.104
Expert opinion (*n* = 550)	*Pro AS*	87 (15.8)	12 (22.2)	64 (14.5)	11 (20.0)	2.20	1.15
	*Contra AS*	158 (28.7)	17 (31.5)	132 (29.9)	9 (16.4)	0.06	4.43^*^
	*Neutral/mixed*	205 (37.3)	11 (20.4)	175 (39.7)	19 (34.5)	7.65^**^	0.54
	*No opinion given*	100 (18.2)	14 (25.9)	70 (15.9)	16 (29.1)	3.45	5.96^*^
Interview/citation of friends/family member/dependent person		46 (5.1)	17 (16.5)	23 (3.2)	6 (7.1)	34.46^***^	n/a
Type of close person (*n* = 46)	*Family*	43 (93.5)	17 (100.0)	20 (87.0)	6 (100.0)	n/a	n/a
	*Friends*	3 (6.5)	1 (5.9)	2 (8.7)	0 (0.0)	n/a	n/a
	*Others*	2 (4.3)	0 (0.0)	2 (8.7)	0 (0.0)	n/a	n/a
Friend/family member/dependent person’s opinion (*n* = 46)	*Pro-AS*	28 (60.9)	9 (52.9)	16 (69.6)	3 (50)	1.15	n/a
	*Contra-AS*	4 (8.7)	2 (11.8)	2 (8.7)	0 (0.0)	n/a	n/a
	*Neutral/mixed*	8 (17.4)	4 (23.5)	2 (8.7)	2 (33.3)	n/a	n/a
	*No opinion given*	6 (13.0)	2 (11.8)	3 (13.0)	1 (16.7)	n/a	n/a
Reported effects of AS on bereaved friends/family		127 (14.0)	26 (25.2)	83 (11.5)	18 (21.4)	14.70^***^	6.68^*^
Pictures		483 (53.3)	47 (45.6)	385 (53.5)	51 (60.7)	2.26	1.56
Statistical data reported		214 (23.6)	25 (24.3)	160 (22.3)	29 (34.5)	0.21	6.29^*^
Cover page		82 (9.1)	5 (4.9)	69 (9.6)	8 (9.5)	2.47	0.00

Values are presented as frequencies with percentages given in parentheses. Symbols (*) indicate significant differences with chi-squared tests or, in case of low frequencies, Fisher’s exact tests; ^*^ *p* < 0.05, ^**^ *p* < 0.01, ^***^ *p* < 0.001 (two-tailed).

n/a = not applicable (Fisher’s exact test).

^1^The degrees of freedom are 1 for chi-squared tests.

### Comparison of period 2 (around the decision of the Constitutional Court of Justice) versus period 1 (before the initiation of legislation change)

Compared to period 1, media items in period 2 were more often viewpoints (*n* = 282, 39.2% vs. *n* = 20, 19.4%) and less often news reports (*n* = 167, 23.2% vs. *n* = 44, 42.7%). A general focus on AS was more common during period 2 (*n* = 611, 85.0% vs. *n* = 48, 46.6%), whereas items with a personal/individual focus were less frequent (*n* = 11, 1.5% vs. *n* = 11, 10.7%). Regarding the content of media items, there was a stronger content focus on palliative care (*n* = 249, 34.6% vs. *n* = 20, 19.4%) and more reflections (pros/cons) of the topic in period 2 (*n* = 708, 98.5% vs. *n* = 89, 86.4%). In contrast, specific considerations about how to prepare for AS (*n* = 149, 20.7% vs. *n* = 33, 32.0%), how to conduct AS (*n* = 106, 14.7% vs. *n* = 31, 30.1%), and about postvention of AS (*n* = 25, 3.5% vs. *n* = 24, 23.3%) were less common in period 2 than in period 1. See **Table 1** for an overview of the frequencies of coding categories of general characteristics in the study periods.

Among the characteristics advised against in media guidelines, media items in period 2 used stigmatizing language more frequently (*n* = 268, 37.3% vs. *n* = 27, 26.2%), more often referred to an epidemic/wave/increase of AS (*n* = 105, 14.6% vs. *n* = 1, 1.0%), and were more often unclear about their reported difficulty level to get AS approved or to carry out AS (*n* = 607, 84.4% vs. *n* = 72, 69.9%).

In contrast, the characteristics advised against in media guidelines that decreased in period 2 as compared to period 1 included monocausal explanations for AS (*n* = 70, 9.7% vs. *n* = 40, 38.8%), the reporting about celebrities considering or dying by AS (*n* = 13, 1.8% vs. *n* = 13, 12.6%), and references to methods of AS in the headline/sub-headline (*n* = 12, 1.7% vs. *n* = 6, 5.8%). Furthermore, media items were less frequently portraying AS as too difficult to get approved or to carry out (*n* = 102, 14.2% vs. *n* = 29, 28.2%). Also, contacts to services that assist in the planning or approval of AS were less frequently included in media items in period 2 compared to period 1 (*n* = 138, 19.2% vs. *n* = 30, 29.1%). See **Table 2** for an overview of the frequencies of coding categories of characteristics advised against in the guidelines in the study periods.

**Table 2:** Reporting of characteristics advised against in the guidelines before, during, and after legislation changes legalizing AS.

Coding categories		Total n (all 3 periods) (*n* = 906)	Period 1 (2017–2019) (*n* = 103)	Period 2 (2020–2021) (*n* = 719)	Period 3 (2022) (*n* = 84)	Comparison of period 1 vs. 2^1^	Comparison of period 2 vs. 3^1^
Characteristics advised against in the guidelines							
Step-by-step description of AS		113 (12.5)	6 (5.8)	74 (10.3)	33 (39.3)	2.05	54.74^***^
Monocausality		129 (14.2)	40 (38.8)	70 (9.7)	19 (22.6)	65.82^***^	12.67^***^
Celebrity		39 (4.3)	13 (12.6)	13 (1.8)	13 (15.5)	n/a^***^	n/a^***^
Substance name		37 (4.1)	5 (4.9)	22 (3.1)	10 (11.9)	n/a	n/a^***^
Romanticization/glorification		464 (51.2)	55 (53.4)	375 (52.2)	34 (40.5)	0.06	4.12^*^
Reference to the method of AS or suicide in the headline/sub-headline		20 (2.2)	6 (5.8)	12 (1.7)	2 (2.4)	n/a^*^	n/a
False myths		325 (35.9)	44 (42.7)	252 (35.0)	29 (34.5)	2.30	0.01
Stigmatizing language		304 (33.6)	27 (26.2)	268 (37.3)	9 (10.7)	4.79^*^	23.48^***^
Epidemic/wave/increase of AS		107 (11.8)	1 (1.0)	105 (14.6)	1 (1.2)	14.91^***^	11.81^***^
Suggesting inevitability		188 (20.8)	19 (18.4)	158 (22.0)	11 (13.1)	0.66	3.57
Difficulty level to get AS approved or to carry out AS	*Yes, AS too difficult to carry out*	158 (17.4)	29 (28.2)	102 (14.2)	27 (32.1)	13.12^***^	17.99^***^
	*No clear valuation*	736 (81.2)	72 (69.9)	607 (84.4)	57 (67.9)	13.22^***^	14.42^***^
	*Yes, AS too easy to carry out*	12 (1.3)	2 (1.9)	10 (1.4)	0 (0.0)	n/a	n/a
Help/contact for AS		195 (21.5)	30 (29.1)	138 (19.2)	27 (32.1)	5.47^*^	7.73^**^

Values are presented as frequencies with percentages given in parentheses. Symbols (*) indicate significant differences with chi-squared tests or, in case of low frequencies, Fisher’s exact tests; ^*^ *p* < 0.05, ^**^ *p* < 0.01, ^***^ *p* < 0.001 (two-tailed).

n/a = not applicable (Fisher’s exact test).

^1^The degrees of freedom are 1 for chi-squared tests.

Some changes were also seen for the recommended content listed in media guidelines. In period 2, possible social pressure that might influence individuals to choose AS was more commonly mentioned than in period 1 (*n* = 211, 29.3% vs. *n* = 6, 5.8%). In contrast, references to crisis intervention services, counseling, or mental health treatment services were less frequent in period 2 (*n* = 17, 2.4% vs. *n* = 9, 8.7%). See **Table 3** for an overview of the frequencies of coding categories of recommended characteristics in the study periods.

**Table 3:** Reporting of recommended characteristics before, during, and after legislation changes legalizing AS.

Coding categories	Total n (all 3 periods) (*n* = 906)	Period 1 (2017–2019) (*n* = 103)	Period 2 (2020–2021) (*n* = 719)	Period 3 (2022) (*n* = 84)	Comparison of period 1 vs. 2^1^	Comparison of period 2 vs. 3^1^
Recommended characteristics						
Debunking false myths	314 (34.7)	37 (35.9)	265 (36.9)	12 (14.3)	0.03	16.96^***^
Social pressure	227 (25.1)	6 (5.8)	211 (29.3)	10 (11.9)	25.65^***^	11.47^***^
Reference to crisis intervention services, counseling, or mental health treatment services	34 (3.8)	9 (8.7)	17 (2.4)	8 (9.5)	n/a^**^	n/a^**^
AS related to crisis or mental health problems	91 (10.0)	16 (15.5)	71 (9.9)	4 (4.8)	3.05	2.32
Alternative(s) to AS	392 (43.3)	38 (36.9)	321 (44.6)	33 (39.3)	2.20	0.88
Different outcome than AS	13 (1.4)	1 (1.0)	12 (1.7)	0 (0.0)	n/a	n/a
Healing bereaved	0 (0.0)	0 (0.0)	0 (0.0)	0 (0.0)	–	–

Values are presented as frequencies with percentages given in parentheses. Symbols (*) indicate significant differences with chi-squared tests or, in case of low frequencies, Fisher’s exact tests; ^*^ *p* < 0.05, ^**^ *p* < 0.01, ^***^ *p* < 0.001 (two-tailed).

n/a = not applicable (Fisher’s exact test).

^1^The degrees of freedom are 1 for chi-squared tests.

### Comparison of period 3 (after the implementation) versus period 2

With the first cases of AS that occurred due to the implementation of the legislation change (period 3), media items featured more background content (*n* = 35, 41.7% vs. *n* = 204, 28.4%) and fewer viewpoints (*n* = 18, 21.4% vs. *n* = 282, 39.2%) than during period 2. Furthermore, media items more often had an individual focus (*n* = 14, 16.7% vs. *n* = 11, 1.5%). There was a stronger focus on the specific preparations for AS (*n* = 33, 39.3% vs. *n* = 149, 20.7%) and related procedures (*n* = 21, 25.0% vs. *n* = 106, 14.7%), whereas a focus on general reflections (pros/cons related to AS) became less frequent compared to period 2 (*n* = 71, 84.5% vs. *n* = 708, 98.5%). See **Table 1** for an overview of the frequencies of coding categories of general characteristics in the study periods.

Regarding characteristics advised against in media guidelines, media items in period 3 more often contained step-by-step descriptions of how to conduct AS (*n* = 33, 39.3% vs. *n* = 74, 10.3%), more frequently provided monocausal explanations for AS (*n* = 19, 22.6% vs. *n* = 70, 9.7%), and more often included references to celebrities considering or dying by AS (*n* = 13, 15.5% vs. *n* = 13, 1.8%). Furthermore, media items more frequently reported the name of the substance used for AS (*n* = 10, 11.9% vs. *n* = 22, 3.1%), and AS was more frequently portrayed as being too difficult to get approved or to conduct (*n* = 27, 32.1% vs. *n* = 102, 14.2%). There was also an increase in items referencing contacts to services supporting the planning or conduction of AS (*n* = 27, 32.1% vs. *n* = 138, 19.2%).

In contrast, characteristics advised against in media guidelines that decreased in period 3 in comparison to period 2 were romanticization/glorification (*n* = 34, 40.5% vs. *n* = 375, 52.2%) and stigmatizing language (*n* = 9, 10.7% vs. *n* = 268, 37.3%). There was also a decrease in items reporting an epidemic or wave of AS (*n* = 1, 1.2% vs. *n* = 105, 14.6%). An overwhelming proportion of media items that described individuals seeking AS used positive adjectives to characterize them in period 3 (*n* = 13, 81.3% vs. *n* = 38, 35.2%). See **Table 2** for an overview of the frequencies of coding categories of characteristics advised against in the guidelines in the study periods.

Some changes were also seen for recommended characteristics: In period 3, it was more common for media items to include a reference to crisis intervention services, counseling, or mental health treatment services (*n* = 8, 9.5% vs. *n* = 17, 2.4%). In contrast, the debunking of false myths about suicide decreased (*n* = 12, 14.3% vs. *n* = 265, 36.9%) as well as references to a possible social pressure that might influence individuals to choose AS to end their lives (*n* = 10, 11.9% vs. *n* = 211, 29.3%). See **Table 3** for an overview of the frequencies of coding categories of recommended characteristics in the study periods.

## Discussion

To the best of our knowledge, this study is the first structured and systematic content analysis of print media items on the topic of AS. We assessed the reporting over a 6-year-long period before as well as during and immediately after a change in legislation allowing for AS.

The analysis revealed some distinct patterns and tendencies in the reporting of AS. Many characteristics that are advised against in media guidelines were very common across the entire observation period. Specifically, a tendency to romanticize AS or portray AS as glorifying was frequent, as was the promulgation of false myths about AS without debunking them. These findings clearly differ from content analyses about the reporting of non-assisted suicide, where these features were typically less frequent: For example, while 51.2% of analyzed media items about AS were categorized to be romanticizing/glorifying AS, a study from the US States of Oregon and Washington analyzing suicide-related reporting between April 2019 and March 2020 found 1.2% of items to glorify/romanticize suicide (18). This proportion was even lower (0.7%) in a study from 2018 covering the time period 2011 to 2014 in Canada (20). Similarly, 33.6% of the analyzed media items in the present analysis about AS used stigmatizing language related to mental health, compared to 9.5% in a study with US data (18), highlighting the relevance of stigma in these media portrayals.

Characteristics recommended in media guidelines were overall less frequent in media items about AS than in previous studies analyzing media reports about non-assisted suicide: For example, 3.7% of the analyzed media items contained a reference to crisis intervention service, counseling, or mental health treatment services, compared to approximately 20% in the study from Oregon and Washington about non-assisted suicide-related reporting (18). Similarly, 10.0% of media items in this study linked AS to crisis situations or mental health problems, compared to 32.5% in that study (18). The proportion was similar to a study covering the period August 2011 to November 2019, which found that 10.9% of items in a Chinese sample of reports linked suicide to mental health problems (31). Importantly, although the studies on (non-assisted) suicide reporting that we have used for comparison included not only print media items like the present study, but also broadcast sources (18), online sources (18, 20), and social media posts from newspaper outlets (31), these large differences between the reporting on AS and non-assisted suicide are unlikely to be solely explained by the media types included in the analysis or cultural differences. These differences might instead reflect different attitudes and opinions on the topics of AS and (non-assisted) suicide. Previous research has found that public attitudes tend to be more supportive of suicide in the case of serious illness as compared to other reasons (32). This might also result in the general support for assisted suicide (as compared to non-assisted suicide), which is often related to serious illness (33, 34).

There is also one study from Austria focusing on non-assisted suicide reporting, which, just like the present analysis, was restricted to print media items and had a strong overlap in terms of specific media outlets included (14). In this previous study, which included data that dates back to the year 2005, even 29.8% of the media items provided a monocausal explanation for suicide, compared to 14.2% in the present analysis. Approximately 17.3% reported on the effects of suicide on bereaved individuals compared to 14.0% in the present sample. Regarding other outcomes than suicide, 8.8% of items in that previous study reported such outcomes, but only 1.4% in the present analysis. Only 2.2% of the news items in that sample enhanced false public myths about suicide, compared to 35.9% in the present analysis. A relation to mental health problems was reported in 19.1% of items (compared to 10.0% in this study), and 5.6% of reports referenced a contact to a support service (compared to 3.8% in the present sample). Overall, this comparison corroborates that the reporting on AS as characterized here is less consistent with media guidelines compared to the reporting on suicide, particularly given that a lot of education efforts have taken place in Austria since 2005, which have very likely resulted in clear improvements on several characteristics, particularly the reporting of help services, which is now the norm in Austrian print media when it comes to the reporting of non-assisted suicide.

Of note, over all three periods, “human/personal rights” was the second-most prevalent reported motivation for AS in the analyzed media items (*n* = 196, 21.6%). In order to explore this in greater depth, we analyzed how this code was related to other characteristics. In the present sample, compared to media items not including human rights as a motivational factor, these media items were more likely to include elements of romanticization (80.1%, *n* = 157 vs. 43.2%, *n* = 307), and they were more likely to report that AS was too difficult to carry out (32.7%, *n* = 64 vs. 13.2%, *n* = 94). These items were also slightly less likely to mention any alternatives to AS compared to other media items (41.8%, *n* = 82 vs. 43.7%, *n* = 310), although this difference was very small. Overall, these patterns seem to corroborate our impression that media portrayals highlighting AS as a human rights issue often, but not always, portrayed individuals who were fighting to get their death provision approved or who needed to go to a different country to carry out AS. It appears plausible to assume that such stories often show some characteristics suggestive of romanticization or glorification, as they relate to human rights activism. From a prevention perspective, it is important to avoid repeated romanticization or glorification of AS, which may influence vulnerable individuals who are unaware of alternatives. Balanced media coverage should neither stigmatize human rights activism nor portray AS as the only option to end suffering.

Some of the changes in reporting characteristics across the three analyzed time periods appear to directly relate to a shift in reporting that corresponds to changes in the legal status of AS. Period 2, the time of most heated debates, was characterized by more sensationalist portrayals as indicated by references to epidemics and waves of AS—in spite of the fact that there were no legal assisted suicides during that time in Austria. These characteristics might reflect concerns about the possible effects of the new legislation. There was also more stigmatization and fewer statements that portrayed AS as too difficult to get approved compared to period 1, which might all reflect the heated and polarized discussions about AS during that period when specific rules and regulations were negotiated. Furthermore, stigmatization as well as romanticization was particularly pronounced in the phase before the implementation of the new legislation, which likely to some extent reflects polarizations in the discussion about the topic.

A tendency to stigmatize AS, which was generally pronounced across the entire observation period, became less prevalent once the first instances of AS in Austria were reported in period 3, and personal stories as opposed to general discussions about the topic of AS became more frequent. This might reflect that, once the legislation was implemented, media items tended to portray personal stories of individuals who conducted AS. Some of these individuals had struggled for quite some time to get their AS approved, and the reporting on their stories might have contributed to a reduction of stigma. The implementation of the legislation was also linked to more step-by-step descriptions of AS and a more frequent mentioning of the name of the substance used for AS, which also likely reflects the reporting of the first specific cases of AS after the legislation change.

From a prevention standpoint, these findings provide insight into what types of content are common in the process leading up to, during, and after legislation changes related to AS. Some, but not all, of the findings are clear matters of concern because they might result in information gaps in the public. First, across the entire period, it was rare to see media items provide references to crisis intervention services, counseling, and mental health treatment. Only a small number of media items linked AS to crisis situations or mental health problems, and only a very small number reported on different outcomes than AS. This is a problem because it is well known that death wishes and suicidal ideation and behaviour are very common after serious diagnoses, and they frequently pass after some time, which makes it important for individuals to be aware of all options they have in the face of suffering and existential fears (35–37). Furthermore, stigmatization as well as romanticization was very common across the entire period, as well as media items that enhanced false myths about suicide. These aspects can easily contribute to misinformation and potential reluctance to seek help (38).

Furthermore, a focus of the reporting on the situation of relatives and friends, either in the form of citations, reports about any effects AS had on them, or on the bereavement process, was rare across the entire observation period. This is concerning because research has shown that family and friends often experience feelings of guilt and are often torn between understanding and support for the death wish of their suffering loved one and hopes that they still might decide against AS (39). These aspects appear grossly underrepresented. Similarly, stories about individuals who considered AS, but ultimately opted for other options, were missing entirely in media reporting on AS.

### Strengths and limitations

A strength of this content analysis is the large number of media items that were included, the systematic assessments of all media items from 11 media outlets across a 6-year period, and the use of a coding system with high intercoder reliability.

There are also some limitations. First, only print media items were included in the content analysis. These reports might differ from items in online media, TV, or radio broadcasts. Future research should also include other media types to assess any differences. Furthermore, only the first year after the implementation of the legislation was included in the analysis due to the availability of data on media reports at the start of this research. Although the periods shortly before and after legislation changes are entirely covered and probably represent times of the highest public attention to AS, it is likely that the quality of reporting will continue to change once AS becomes more established. It is therefore important to assess longer-term changes after the implementation beyond the first year. Also, the “difficulty level to get AS approved or to carry out AS” in combination with potential reported obstacles to carry out AS needs further investigation, particularly regarding the specific obstacles and challenges that are highlighted in the media reports. Finally, it is important to assess how the reporting on AS continues to develop over time, as well as how reporting on AS differs between countries where AS has recently been legalized and countries with a long history of legal options for AS.

### Conclusion

This content analysis of newspaper items from Austrian daily newspapers based on media guidelines for the reporting of suicide revealed that stigmatization as well as romanticization/glorification was common in the reporting of AS and most pronounced in the phase immediately before the implementation of the new legislation. Other aspects, particularly the enhancement of false public myths about suicide, and a prevalent tendency to portray AS as inevitable were of concern as well. A link of AS to mental health problems or links to resources for crisis intervention or counseling were very rare across the observation period.

Media guidelines for the reporting on suicide appear to be a helpful tool to assess characteristics of the reporting of AS, but adaptations of the original guidelines in terms of framing and specific codes are necessary in order to ensure the characteristics are applicable to AS. Future research might use the present study as a basis to add further codes that are relevant but are not part of media guidelines for the reporting on suicide and have not been captured in this analysis. Ultimately, this might result in the development of a separate set of media guidelines for the portrayal of AS in the future.

In the meantime, we recommend considering media guidelines for reporting on suicide in a similar way as in the present analysis, i.e., use some of the codes that we have applied to analyze media reporting on AS to support balanced reporting about this complex topic and help make sure that the public is better informed about AS. Based on this analysis, a focus on media reporting in prevention work related to AS appears necessary throughout the process of discussing and implementing new legislation as well as in the post-implementation phase, and particularly, the phase immediately preceding the implementation requires the strongest attention in order to help ensure that the public discourse is well-informed and balanced.

## Data Availability

The data analyzed in this study was obtained from the Austrian Press Agency. This data is copyright the Austrian Press Agency and available on purchase. Requests to access the datasets should be directed to APA Online Manager (https://www.aomweb.apa.at/).

## References

[1] Meier DE Emmons CA Wallenstein S Quill T Morrison RS Cassel CK. A national survey of physician-assisted suicide and euthanasia in the United States. N Eng J Med. (1998) 338:1193–201. doi: 10.1056/NEJM199804233381706, PMID: 9554861

[2] Paterson C. On clarifying terms in applied ethics discourse: Suicide, assisted suicide, and euthanasia. Int Philos Q. (2003) 43:351–8. doi: 10.5840/ipq200343321

[3] Picón-Jaimes YA Lozada-Martinez ID Orozco-Chinome JE Montaña-Gómez LM Bolaño-Romero MP Moscote-Salazar LR. Euthanasia and assisted suicide: An in-depth review of relevant historical aspects. Ann Med Surg (Lond). (2022) 75:103380. doi: 10.1016/j.amsu.2022.103380, PMID: 35242326 PMC8857436

[4] Riisfeldt TD. Overcoming conflicting definitions of “Euthanasia,” and of “Assisted suicide,” Through a value-neutral taxonomy of “End-of-life practices. J Bioeth Inq. (2023) 20:51–70. doi: 10.1007/s11673-023-10230-1, PMID: 36729348 PMC10126086

[5] Mroz S Deliens L Cohen J. Chambaere K Developments under assisted dying legislation: the experience in Belgium and other countries. Dtsch Arztebl Int. (2022) 119:829–35. doi: 10.3238/arztebl.m2022.0378

[6] Sterbeverfügungsgesetz. [Death Provision Law] (2022). Available online at: https://www.ris.bka.gv.at/NormDokument.wxe?Abfrage=Bundesnormen&Gesetzesnummer=20011782&Artikel=&Paragraf=6&Anlage=&Uebergangsrecht (Accessed July 24, 2025).

[7] Masel EK. Perspective: legal, ethical, and medical perspectives of the landscape of assisted suicide in Austria. Wiener Klin Wochenschr. (2024) 136:380–1. doi: 10.1007/s00508-024-02344-2, PMID: 38530423

[8] Tomandl G Kapitany T Stein C Sonneck G Niederkrotenthaler T Marboe G. Leitfaden zur Berichterstattung über Suizid. Guidel News Rep Suicide. (2025). Available online at: https://static1.squarespace.com/static/638a6e7a684c250c7777e1e6/t/67ecfd9d6776352ccadaf555/1743584673471/Leitfaden+zur+Berichterstattung+ueber+Suizid_DRUCK_2025.pdf (April 17, 2025).

[9] Bodas M Ziv A Rubin C Oberman B Tawil Y Shaulov A. Polarization in public attitudes toward end-of-life decisions in Israel–A cross-sectional study. Palliat Support Care. (2024) 22:1615–22. doi: 10.1017/S1478951523000780, PMID: 37365823

[10] Walker S Egan R Young J Jaye C Jackson C. A citizens’ jury on euthanasia/assisted dying: Does informed deliberation change people’s views? Health Expect. (2020) 23:388–95. doi: 10.1111/hex.13008, PMID: 31820555 PMC7104650

[11] Klesse R Teising M Lewitzka U Bäurle P Ciompi L Fiedler G. Assistierter Suizid und Autonomie–ein Widerspruch? [Assisted suicide and autonomy: A contradiction]? Gießen: Psychosozial-Verlag. (2022) 45:97–133. doi: 10.30820/0171-3434-2022-3

[12] McCombs M Valenzuela S. Setting the Agenda. 3rd Edition. Cambridge, UK: Polity Press (2020).

[13] WHO (World Health Organization). Preventing suicide: a resource for media professionals, update 2023. Geneva, Switzerland: World Health Organization (2023).

[14] Niederkrotenthaler T Voracek M Herberth A Till B Strauss M Etzersdorfer E. Role of media reports in completed and prevented suicide: Werther v. Papageno effects. Br J Psychiatry. (2010) 197:234–43. doi: 10.1192/bjp.bp.109.074633, PMID: 20807970

[15] Fu KW Yip PSF. Changes in reporting of suicide news after the promotion of the WHO media recommendations. Suicide Life Threat Behav. (2008) 38:631–6. doi: 10.1521/suli.2008.38.5.631, PMID: 19014313

[16] McTernan N Spillane A Cully G Cusack E O’Reilly T. Arensman E Media reporting of suicide and adherence to media guidelines. Int J Soc Psychiatry. (2018) 64:536–44. doi: 10.1177/0020764018784624, PMID: 29972096

[17] Niederkrotenthaler T Schacherl R Till B. Communication about suicide in YouTube videos: content analysis of German-language videos retrieved with method-and help-related search terms. Psychiatry Res. (2020) 290:113170. doi: 10.1016/j.psychres.2020.113170, PMID: 32526517

[18] Niederkrotenthaler T Laido Z Gould M Lake AM Sinyor M Kirchner S. Associations of suicide-related media reporting characteristics with help-seeking and suicide in Oregon and Washington. Aust N Z J Psychiatry. (2023) 57:1004–15. doi: 10.1177/00048674221146474, PMID: 36579678

[19] Pirkis J Blood RW Beautrais A Burgess P. Skehan J Media guidelines on the reporting of suicide. Crisis. (2006) 27:82–7. doi: 10.1027/0227-5910.27.2.82, PMID: 16913330

[20] Sinyor M Schaffer A Nishikawa Y Redelmeier DA Niederkrotenthaler T Sareen J. The association between suicide deaths and putatively harmful and protective factors in media reports. CMAJ. (2018) 190:E900–7. doi: 10.1503/cmaj.170698, PMID: 30061324 PMC6066401

[21] Till B Niederkrotenthaler T. Surfing for suicide methods and help: Content analysis of websites retrieved with search engines in Austria and in the United States. J Clin Psychiatry. (2014) 75:886–92. doi: 10.4088/JCP.13m08861, PMID: 25099284

[22] Till B Braun M Gahbauer S Reisinger N Schwenzner E Niederkrotenthaler T. Content analysis of suicide-related online portrayals: changes in contents retrieved with search engines in the United States and Austria from 2013 to 2018. J Affect Disord. (2020) 271:300–9. doi: 10.1016/j.jad.2020.03.063, PMID: 32479330

[23] Utterson M Daoud J Dutta R. Online media reporting of suicides: analysis of adherence to existing guidelines. BJPsych Bull. (2017) 41:83–6. doi: 10.1192/pb.bp.115.052761, PMID: 28400965 PMC5376723

[24] Verein Arbeitsgemeinschaft Media-Analysen Wien. MA 23/24 (Media-Analyse 2023/2024) [MA 23/24 (Media Analysis 2023/2024)] (2024). Available online at: https://www.media-analyse.at/table/4144 (Accessed April 17, 2025).

[25] Mayring P. Qualitative Content Analysis: A Step-by-Step Guide. Thousand Oaks: Sage (2022).

[26] Niederkrotenthaler T Ladinser E Arbeitsgruppe Stigmafrei. Stigmafrei: Empfehlungen zur Berichterstattung über psychische Erkrankungen [Stigma-free: Recommendations for reporting on mental illness] (2021). Available online at: https://www.stigma-frei.at/literatur-downloads/ (Accessed 24 July, 2025).

[27] Freelon D. ReCal2, Reliability for 2 Coders . Available online at: https://dfreelon.org/utils/recalfront/recal2/ (Accessed April 17, 2025).

[28] Hallgren KA. Computing inter-rater reliability for observational data: an overview and tutorial. Tutor Quant Methods Psychol. (2012) 8:23. doi: 10.20982/tqmp.08.1.p023, PMID: 22833776 PMC3402032

[29] Krippendorff K. Content analysis: An introduction to its methodology. 4th Edition. Thousand Oaks: Sage (2018).

[30] Martínandrés A Tejedor IH. On conditions for validity of the approximations to Fisher’s exact test. Biom J. (1997) 39:935–54. doi: 10.1002/bimj.4710390806

[31] Lai K Li D Peng H Zhao J He L. Assessing suicide reporting in top newspaper social media accounts in China: content analysis study. JMIR Ment Health. (2021) 8:e26654. doi: 10.2196/26654, PMID: 33983127 PMC8160790

[32] Tong Y Phillips JA. Understanding changes in attitudes toward suicide between 1980s and 2010s in the United States. Soc Sci Q. (2018) 99:1585–98. doi: 10.1111/ssqu.12522

[33] Pentaris P Jacobs L. UK public’s views and perceptions about the legalisation of assisted dying and assisted suicide. Omega (Westport) OMEGA-Journal Death Dying. (2022) 86:203–17. doi: 10.1177/0030222820947254, PMID: 32746764

[34] Stolz E Mayerl H Gasser-Steiner P Freidl W. Attitudes towards assisted suicide and euthanasia among care-dependent older adults (50+) in Austria: the role of socio-demographics, religiosity, physical illness, psychological distress, and social isolation. BMC Med Ethics. (2017) 18:71. doi: 10.1186/s12910-017-0233-6, PMID: 29212490 PMC5719645

[35] Monforte-Royo C Villavicencio-Chávez C Tomás-Sábado J Mahtani-Chugani V Balaguer A. What lies behind the wish to hasten death? A systematic review and meta-ethnography from the perspective of patients. PloS One. (2012) 7:e37117. doi: 10.1371/journal.pone.0037117, PMID: 22606338 PMC3351420

[36] Monforte-Royo C Crespo I Rodríguez-Prat A Marimon F Porta-Sales J Balaguer A. The role of perceived dignity and control in the wish to hasten death among advanced cancer patients: a mediation model. Psychooncology. (2018) 27:2840–6. doi: 10.1002/pon.4900, PMID: 30251342

[37] Ohnsorge K Rehmann-Sutter C Streeck N Gudat H. Wishes to die at the end of life and subjective experience of four different typical dying trajectories. A qualitative interview study. PloS One. (2019) 14:e0210784. doi: 10.1371/journal.pone.0210784, PMID: 30653575 PMC6336242

[38] Niederkrotenthaler T Reidenberg DJ Till B Gould MS. Increasing help-seeking and referrals for individuals at risk for suicide by decreasing stigma: the role of mass media. Am J Prev Med. (2014) 47:235–43. doi: 10.1016/j.amepre.2014.06.010, PMID: 25145745

[39] Marek F Woehrle J Oexle N. Angehörige, die einen assistierten Suizid begleiten: ein Kommentar zum Forschungsstand. [Relatives accompanying an assisted suicide—a commentary on the state and need for research. Suizidprophylaxe. (2022) 49:125–7.

