# Supplementary material for: Media portrayals of assisted suicide before, during, and after legalization changes: content analysis of the reporting in Austrian newspapers
Source: Front Psychiatry. 2025 Sep 18;16:1617602. doi: 10.3389/fpsyt.2025.1617602 (PMC12488708; doi:10.3389/fpsyt.2025.1617602)
Supplement: Supplementary file 1 [file DataSheet1.pdf]

Supplementary materials for the article

Supplementary Table S1. *Coding scheme.*

Codebook

**Coding Category**	**Code Description & Coding Examples**	**Codes**	**Krippendorff's Alpha (α)**
**General Characteristics**			
Article code	Media items are numbered consecutively for each year in the analysis period, followed by the ID of the respective newspaper (see below, #4) e.g., 2022_27_SN.<br><br>Newspaper IDs: Der Standard - ST; Die Presse - PR; Heute - HE; Kleine Zeitung - KL; Kurier - KU; Kronen Zeitung - KR; Oberösterreichische Nachrichten - ON; Salzburger Nachrichten - SN; Tiroler Tageszeitung - TT; Vorarlberger Nachrichten - VN; Wiener Zeitung - WZ.	Open	
Publication date	DD.MM.YYYY (Example: 02.06.2023)	(Open field)	
Media source		- Der Standard<br>- Die Presse<br>- Heute<br>- Kleine Zeitung<br>- Kurier<br>- Kronen Zeitung<br>- Oberösterreichische Nachrichten<br>- Salzburger Nachrichten<br>- Tiroler Tageszeitung<br>- Vorarlberger Nachrichten<br>- Wiener Zeitung	
Title of the article	Full title of the media item.	Open	
Type of content	Please select the most appropriate type of media item.	- News	1.00

	Code reports about interviews as interview even if they do not fully follow an interview style.<br><br>If report is related to a new and timely event that occurred recently -> code news.<br>If report is related to background analyses or considerations related to a recent news event -> code background.	- Background<br>- Comment (reader's view/editor's view)<br>- Interview<br>- Other (Open field)	
Killing on request	Is killing on request a topic in the item? Code positive even if it is only covered in a short section of the media item.<br>Definitions:<br>Assisted suicide: person received medication for own use<br>Active euthanasia: Person gets lethal injection by another person<br>Passive euthanasia: Providing high dosages of pain medication for pain relief; turning off life-prolonging procedures.	- yes<br>- no	1.00
Article focus (individual vs general)	* If the report primarily covers stories about (a) specific individual(s), code "individual focus".<br><br>* If the story is primarily about the phenomenon of AS without any individual focus, code "general focus".<br><br>* If both are present and in some balance (i.e., no clear dominance of either individual components or general components), code "mixed focus".	- Individual focus<br>- General focus<br>- Mixed focus	1.00
Description of character of a person considering AS or dying by AS	Only applicable if code "Article focus (individual vs general)" is coded as "Individual focus" or "Mixed focus".<br>Description of a person who is either considering or dying by AS beyond socio-demographics and illness. Reporting of mental illness (e.g., "he was ill", "depressed) does not qualify as a character description.	- Good<br>- Bad<br>- Mixed	0.93

	Extraordinary skills qualify as well, if they are highlighted as such.<br><br>Code "Mixed" if good and bad descriptors are present in a balanced way, or if there is some character description, but it is not clear if the description is positive or negative.<br><br>NOTE THE DIFFERENCE IN EXAMPLES:<br>The statement “He was a happy person, always cheerful” qualifies as character description, but “He was happy and smiling before his AS...” is not a character description, because it refers to a state (mood) rather than a trait description.<br><br>EXAMPLES:<br>Examples: “He was a terrible man”, “He never did anything right” or “She was...a loving mother”, “She was gentle”.<br><br>Any reported criminal acts have to be taken into account for this code.<br>If a positive character description comes up, but at the same time the person is portrayed as criminal, the combination would qualify for the code category “mixed”.		
Content focus	Code the main focus area(s) in the media item. Please code any focus area relevant in the media item. To qualify as a focus, a significant portion of the text needs to be about the specific focus, normally at least one paragraph.<br>- Preparation is focused on anything that needs to be done before conducting an AS (e.g., assessments with physicians; setting up a death provision [Sterbeverfügung;] collection of lethal substance from pharmacy).	- Preparation of AS	0.93
		- Process of carrying out AS	1.00
		- Postvention AS	1.00

	- Process of carrying out AS is everything that focusses on the act of AS after finalized preparations (e.g., taking the medication)<br>- Reflection includes pros / cons of AS<br>- Postvention includes all measures and behaviours that aim to provide assistance to bereaved individuals after AS. To qualify as postvention, these measures need to follow established strategies for postvention.<br>-Suicide Prevention: focus is on suicide prevention<br>EXAMPLES: Reporting about support groups after AS qualify as postvention.<br>-The provision of suicide prevention services / contacts at the end of an item does not qualify as “focus suicide prevention”	- Palliative Care	1.00
		- Suicide prevention	1.00
		- Reflection (pro / con AS)	0.91
		- Other	1.00
Motivations	Code the main motivation(s) for AS as portrayed in the media item.<br>Code all that apply.<br>If the answer is "other", please insert the specific motivation.<br><br>If the media item focuses on patients in palliative care, this qualifies for a motivation related to “somatic ilness.”	- Any illness, unspecified if mental /somatic	0.94
		- Mental illness (other than dementia)	1.00
		- Somatic illness	0.89
		- Dementia	1.00
		- Age / life weariness	1.00
		- Human rights / personal rights	1.00
		- Isolation / loneliness	1.00
		- Burden to others	1.00
		- Alternative to non-assisted suicide	1.00
		- Pain & suffering	1.00
		- Other	1.00
		- No motivation	1.00
Country situations	Check all countries which are represented in the media item.	- Austria	1.00
		- Germany	1.00

	If the situation is obviously domestic code “Austria” even in the absence of an explicit label.<br>Check all that apply.<br>For code category "other country", please insert country name.	- Switzerland	1.00
		- Belgium	1.00
		- Netherlands	1.00
		- Spain	1.00
		- Portugal	1.00
		- Italy	1.00
		- Other country<br>(+ open field)	1.00
		- No specific country	1.00
Interview / citation of expert	Code if the media item includes an interview or a direct or indirect citation of an expert. An expert is a person who works/has her or his profession in a field that is affected by the concept of AS.	- Yes<br>- No	1.00
Type of expert	Only applicable if “Interview/citation of expert” is coded positive.<br>Check all that apply.<br>In the case of an expert group not on the list please tick "other" and insert profession.<br><br>If the speciality of a medical doctor is not known code as “medical doctor other than psychiatrist”.<br><br>If the person assisting in suicide is not affiliated with a pro-AS organization code as “suicide assistant not from an organization”. If there is any pro-AS organization mentioned in the same media item which might be related to the assisting person code as “suicide organization”, even if the link is not explicitly made in the report.	- Representative of ethical body	1.00
		- Legal expert	1.00
		- Medical doctor other than psychiatrist / pall care physician or unspecified	1.00
		- Psychiatrist	1.00
		- Palliative care physician	1.00
		- Suicide assistant not from an organization	1.00
		- Psychologist / psychotherapist	1.00

		- Suicide organization	1.00
		- Funeral director	1.00
		- Author / editor / media professional	1.00
		- Politician	1.00
		- Others or unspecified (+ open field)	0.94
Expert opinion	If two or more experts are interviewed/cited, decide on the combination of opinions portrayed in the item overall.	- Pro AS<br>- Contra AS<br>- Neutral/mixed<br>- No opinion given	0.96
Interview/citation of friends/family member/dependent person	This includes any individuals with some kind of close relationship with the person considering or dying from AS who are not coded as “experts”.	- Yes<br>- No	1.00
Type of close person	Only applicable if “Interview/citation of friends/family member/dependent person” is coded positive.<br>If the answer is "Others", please specify what kind of relationship exists with the person considering or dying from AS.	- Family	1.00
		- Friends	1.00
		- Others (+ open field)	1.00
Friend/family member/dependent person opinion	Only applicable if “Interview/citation of friends/family member/dependent person” is coded positive.<br>Please specify the main opinion of the stated/interviewed friend/family member/dependent person.<br>In case of several or conflicting opinions, please assess the overall tendency. In case of a balance, code “neutral / mixed”.	- Pro AS<br>- Against AS<br>- Neutral / mixed<br>- No opinion given	1.00

	If the dependent / friend expresses mainly the opinion of support/freedom regarding the wish for AS code "pro AS".<br>- If the dependent / friend expresses the opinion of rejection/fear/ regarding AS or feels desperate because of AS, code “against AS”.<br>- If the dependent / friend expresses rather neutral feelings code “Neutral / mixed”.<br>- If no opinion is given, code “No opinion given.”<br><br>If two or more persons from friend/family member/dependent person are interviewed/cited, assess the combination of opinions reflected in the media item overall.		
Reported effects of AS on bereaved friends / family	Any effects of AS on close person(s). Relatives or friends of the decedent or any other persons privately involved in the act of AS or in the investigation of AS are defined as friends/family.	- Yes<br>- No	1.00
Pictures	Code positive if there are any pictures included in the media item.	- Yes<br>- No	1.00
Statistical data reported	A quantitative summary/analysis of any AS data or of suicide or morbidity data (e.g., depression) or social data (e.g., AS requests) related to AS.<br><br>NOTES:<br>A single sentence e.g., related to current case numbers is sufficient to qualify for this code.	- Yes<br>- No	1.00
Cover Page	Does the media item have a headline/teaser on the front page or is even the entire item on the front page?	- Yes<br>- No	1.00

Characteristics advised against in guidelines			
Step-by-step description of AS	To qualify for this code, the media item needs to report at least two consecutive steps in the planning or in the carrying out of AS.	- Yes<br>- No	1.00
Monocausality	Exactly one possible motive, cause, or trigger of AS is reported.<br>The cause needs to have some specificity, e.g., "unbearable suffering [in German: unerträgliches Leid] does not qualify as "monocausal" as the nature / cause of suffering remains entirely unclear. In contrast, "unbearable pain" would be more specific and would therefore potentially qualify.<br><br>If there are several different factors (motives, causes, or triggers) mentioned in the report these factors should be taken into account even if they are not explicitly labelled as a motive. Thus, monocausality should be coded negative in these instances.<br><br>If the media item describes the requirements for AS according to the law, this does not qualify as monocausal.<br><br>EXAMPLES: If a person featured in the item says: "The only reason for my wish for AS was depression", but the text subsequently mentions other causes/motives/triggers, e.g., unemployment, the report does not qualify as monocausal in spite of the fact that unemployment is not explicitly labelled as being causal.<br><br>If one specific cause/trigger is mentioned and some unspecific statement about further problems that were present (e.g., "he	- Yes<br>- No	0.93

	was depressed and had other problems"), this would not qualify as monocausal either.		
Celebrity	Code positive if individuals considering or dying by AS are celebrities. Celebrities can be local celebrities (e.g., a local professional football player) or international celebrities. Individuals who become celebrities due to their engagement for or against AS can also be coded here.<br><br>Stories about thoughts regarding AS from relatives or friends of the celebrity qualify as well if they are reported based on the celebrity status of their relative / friend.	- Yes<br>- No	1.00
Substance Name	Code if the substance name of any substance involved in AS is provided.<br><br>If the answer is "Yes", please state the name of the substance in the blank field.	- Yes (+ open field)<br>- No	1.00
Romanticization / glorification	Code any aspect that might glorify or romanticize the phenomenon of AS, individuals considering AS or dying by AS or portray them as a hero, even if implicit only or subtle.<br><br>"Allow dying with dignity" qualifies as romanticization if the term "dying with dignity" is used as a generic term for AS, because this would imply that AS is equivalent to the broader concept of "dying with dignity" which includes other options beyond AS as well.<br><br>If the media item, however, states that AS allowed a specific individual to die with dignity, this would not qualify as a romanticization.	- Yes<br>- No	1.00

	The reporting that an individual suffered unbearable pain does not qualify as glorification.<br><br>Examples:<br>- Independence until death [Unabhängigkeit bis zum Tod]<br>“Also his last choice is his own choice” ["Auch beim letzten Weg will XX selbst entscheiden"]<br><br>- Now they will be reunited after death. [“Jetzt werden sie nach dem Tod wieder vereint”]		
Reference to method of AS or suicide in headline / sub-headline	Reference to specific method in the headline.	- Yes<br>- No	1.00
False myths	The media item enhances a false public myth on AS. This includes both explicit (wrong) statements and implicit statements that might make one of the myths below appear to be correct.<br><br>The following myths qualify:<br>AS is primarily and exclusively an<br>- act of free will;<br>- good for the environment (family / friends); or<br>- an alternative for non-assisted suicide<br>Also myths such as<br>-“once suicidal , always suicidal” ;<br>-“patients in palliative care typically / most of the time want to die”<br>-“nothing can be done to prevent suicide”; or<br>-“those who speak about AS (or suicide) won’t do it”<br>qualify here.<br><br>Statements that it	- Yes<br>- No	1.00

	-“it is nearly impossible to receive AS after the legislation change in 2022 in Austria”, or that<br>-“Death wishes are always suicide wishes” qualify as well.		
Stigmatizing language	Check if the story uses stigmatizing language related to AS, suicide and / or mental health problems such as e.g., “Selbstmord” [“self-murder”]; "to commit suicide", "an (un)successful suicide or AS, "a failed attempt", or colloquial terms used to devaluate suicide decedents or suicidal individuals (e.g., “crazy”, “dumb”, “silly”, etc.).<br>If unclear, please consult the "Stigmafrei Guidelines": https://www.stigma-frei.at/empfehlungen/<br><br>If current discussions about AS are portrayed as being directly related to euthanasia in the context of national socialism or reflecting the same underlying attitude, code as stigmatizing.<br><br>NOTE:<br>A sheer negative appraisal of AS (e.g., a statement that AS can be perceived selfish) without devaluing an individual considering AS or offering assistance in AS, and without stigmatizing the act of AS, does not qualify for this code.	- Yes<br>- No	0.92
Epidemic/wave/increase of AS	Wording that describes AS as a spreading phenomenon, involving more and more individuals, or an extraordinarily high number of individuals, or concerns about such increases. Includes expressions such as “waves” of AS, “epidemics”, “series” of AS, “flooding [“*Schleusentor geöffnet*”]; floodgates [“*Dammbruch*”]; “highest rate” of AS, “alarming trend”, “a tremendous rise in AS” and similar wording.<br><br>NOTE:	- Yes<br>- No	1.00

	It does not matter if the message is factually correct or not to assess this code.<br>Reporting on an increase in rates of AS without any sensationalist wording does not qualify. Similarly, a message of “higher rates than last year” does not qualify.		
Suggesting inevitability	The story contains wording suggesting inevitability of AS. This includes statements suggesting that AS is not preventable or cannot be avoided.<br>A media item that highlights that “everything possible” was done, but still somebody died by AS, does not qualify for this code because this creates the impression that the outcome might have been different.<br><br>If a person is portrayed to have considered AS for a very long time even before it was legalized and now they are finally planning or following through, in the absence of other statements suggesting that this was (at least at any point in the process) not etched in stone, this would qualify as “inevitable”.<br><br>If AS is portrayed as absolutely necessary to allow for a death in dignity, without adding alternatives ways for dying in dignity, also code positive.<br><br>EXAMPLES<br>”There was no way to stop it” or ”it was going to happen no matter what”.	- Yes (+ open field)<br>- No	1.00
Difficulty level to get AS approved or to carry out AS	Code if the media item portrays AS to be easy or difficult to get approved or to realize. Difficulties reported are often related to the approval of AS.<br>Also implicit valuations, e.g. an expert saying that AS should be legalized or should be considered in a specific case, qualify for the assessment of this code. For example, before the	- Yes, AS portrayed as (too) difficult to carry out<br>- No (clear) valuation in terms of difficulty<br>- Yes, AS portrayed as (too) easy to carry out	0.95

	legislation during the time when AS was illegal (i.e., before the rule of court), saying "society should consider AS" means that it is "too difficult" based on the status quo at this point in time.		
Help/contact for AS	Code if there is a contact to persons or organizations (name, email, address, phone number, website) assisting in AS is provided. For example, a reference to an organization, to an expert, etc., that will support individuals in the process of getting approval of a death provision.<br><br>Please note that pro-AS organizations informing individuals about AS qualify for this code.	- Yes (+ open field)<br>- No	1.00
**Recommended characteristics**			
Debunking false myths	Using the definition of "public myths", this includes stories that explicitly or implicitly debunk any of the listed myths (see list above, myths enhanced).<br>Also code positive if a person is reported to have been able to get AS in Austria because this debunks the myth that it is not possible to carry out an AS in Austria.	- Yes<br>- No	0.83
Social pressure	Code positive if the media item brings up that social pressure to conduct AS on chronically ill or older individuals is or might become a problem at some point.<br><br>EXAMPLE:<br>"The aunt put a lot of pressure on him to think about AS."	- Yes<br>- No	0.93
Reference to crisis intervention services, counselling or mental health treatment services	Code if any contact (email, phone number, address or website with contacts) is provided to assist individuals considering AS or suicidal or in crisis or bereaved by suicide or AS.	- Yes<br>- No	1.00

	Do not code here contacts to organizations and individuals supporting in the planning or conduction of AS as this is coded elsewhere.<br><br>Only naming a specific psychologist, medical doctor or similar, without providing further contact details, does not qualify for this code.		
AS related to crisis or mental health problems	The story reports about a connection/link between mental health and AS.<br>The relationship between mental disorders/mental health problems and AS or suicidality is acknowledged, either at the level of an individual considering (or dying from) AS or in general terms.<br>The link between mental health and AS can also be indirect, e.g., the story might report about support for mental health problems and then describe it as a means of prevention of AS.<br><br>NOTE:<br>Stigmatizing wording such as "crazy" do not qualify as a reference to mental health.	- Yes<br>- No	1.00
Alternative(s) to AS	This might include a specific action taken by an individual instead of AS; a suggestion /advice to seek help; or guidance on how to overcome suicidal thoughts in the context of serious illness, e.g., getting palliative care.<br><br>NOTES:<br>A reference to the crisis intervention center or another support service at the end of the text does not qualify. The alternative needs to be a part of the narrative of the story rather than being placed at the end of the text.	- Yes (+ open field)<br>- No	1.00

	Writing about palliative care in a media item about AS means that an alternative is given (even if palliative care is not explicitly labelled as an alternative).<br><br>If the “alternative” is very general and unspecific (i.e., without any likely practical use to someone in the state of considering AS), do not code as alternative.<br><br>Only code positive, if the alternative is not portrayed as being ineffective or unavailable. E.g., if a media item emphasises that there are many cases of AS because there are no palliative care units, this would not qualify as an “alternative”, because the alternative is reported as “not available”.		
Different outcome than AS	The story reports on a person considering AS, but taking other action not involving suicide or AS.<br><br>NOTES:<br>Examples: Someone calling a suicide hotline/getting help; getting palliative care support.	- Yes<br>- No	1.00
Healing bereaved	The story reports a healing process of a person bereaved by AS. Any personal story of coping after AS should code positive here, even if the “positive outcome” is not explicitly stated.	- Yes<br>- No	1.00

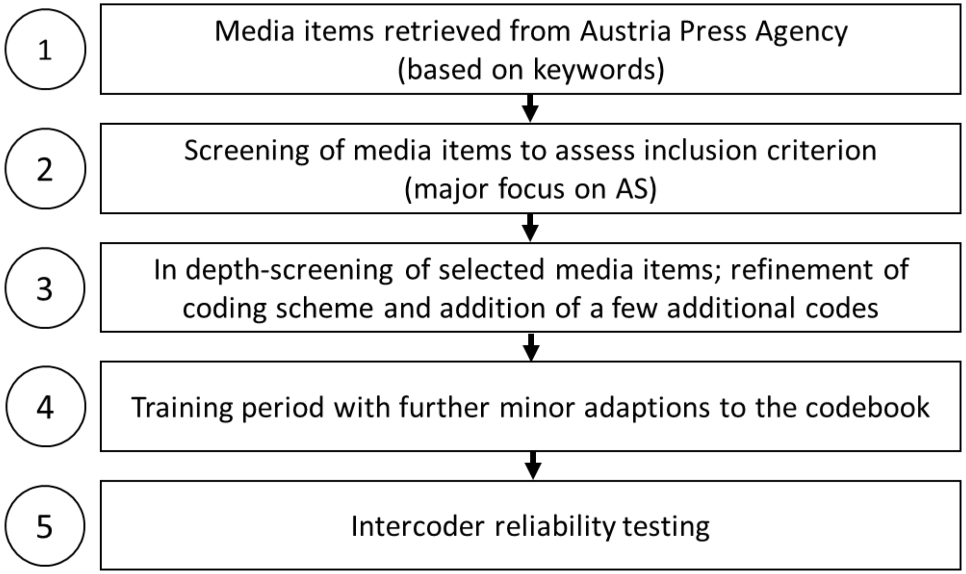

Supplementary Figure S1. *Overview of coding process.*
